# Association of Intraventricular Hemorrhage and Death With Tocolytic Exposure in Preterm Infants

**DOI:** 10.1001/jamanetworkopen.2018.2355

**Published:** 2018-09-21

**Authors:** Gaëlle Pinto Cardoso, Estelle Houivet, Laetitia Marchand-Martin, Gilles Kayem, Loïc Sentilhes, Pierre-Yves Ancel, Elsa Lorthe, Stéphane Marret

**Affiliations:** 1 Department of Neonatal Pediatrics and Intensive Care, Neuropediatrics and Rehabilitation Center, Reference Centre for Learning Disabilities of the Child, Rehabilitation Centre, Rouen University Hospital–Charles Nicolle Hospital, Rouen, France; 2 Institut National de la Santé et de la Recherche Medicale (INSERM) U1245, NEOVASC Team, Research and Biomedical Innovation Institute, Rouen Medical School, Normandy University, Rouen, France; 3 Department of Biostatistics, Rouen University Hospital, Rouen, France; 4 INSERM U1153, Epidemiology and Statistics Sorbonne Paris Cité Center, Obstetrical, Perinatal and Pediatric Epidemiology Team, Maternité Port-Royal, Paris Descartes University France, Département Hospitalo-Universitaire Risk in Pregnancy, Paris, France; 5 Unité de Recherche Clinique, Centre d’Investigation Clinique P1419, Cochin Hotel-Dieu Hospital, Assistance Publique–Hôpitaux de Paris, Paris, France; 6 Department of Obstetrics and Gynecology, Armand Trousseau Hospital, Paris, France; 7 Sorbonne Universités, Université Pierre and Marie Curie Paris 06, Paris, France; 8 Department of Obstetrics and Gynecology, Bordeaux University Hospital, Bordeaux, France

## Abstract

**Importance:**

No trials to date have demonstrated the benefits of tocolysis on death and/or neonatal morbidity in preterm infants; tocolytics may affect the fetal blood-brain barrier.

**Objectives:**

To assess the risks associated with tocolysis in women delivering prematurely as measured by death and/or intraventricular hemorrhage (IVH) in preterm infants and to compare the association of calcium channel blockers (CCBs) nifedipine and nicardipine hydrochloride vs atosiban used for tocolysis with death and/or IVH.

**Design, Settings, and Participants:**

The French 2011 EPIPAGE-2 (Enquête Épidémiologique sur les Petits Âges Gestationnels) cohort was limited to mothers admitted for preterm labor without fever, who delivered from 24 to 31 weeks of gestation from April 1 through December 31, 2011. Groups of preterm infants with vs without tocolytic exposure and groups with atosiban vs CCB exposure were compared. Data analysis was performed from June 7, 2014, through September 3, 2017.

**Exposures:**

Tocolytics.

**Main Outcomes and Measures:**

The primary outcome was a composite of death and/or IVH in preterm infants. Secondary outcomes included death, IVH, and a composite of death and/or grades III to IV IVH.

**Results:**

A total of 1127 mothers (mean [SD] age, 25.5 [6.0] years) experienced preterm labor and gave birth to 1343 preterm infants with a male to female ratio of 1.23 and mean (SD) gestational age of 27 (2.5) weeks. Of these, 789 mothers (70.0%) received tocolytics; 314 (39.8%) received only atosiban, and 118 (15.0%) received only a CCB. In the first analysis, the primary outcome (death and/or IVH) was not significantly different in preterm infants with vs without tocolytic exposure (183 of 363 [50.4%] vs 207 of 363 [57.0%]; relative risk [RR], 0.88; 95% CI, 0.77-1.01; *P* = .07). The secondary outcome (death and/or grades III-IV IVH) was significantly lower in preterm infants with vs without tocolytic exposure (92 of 363 [25.3%] vs 118 of 363 [32.5%]; RR, 0.78; 95% CI, 0.62-0.98; *P* = .03). Other outcomes did not differ significantly. In the secondary analysis, death and/or IVH was not significantly different in preterm infants with atosiban vs CCB exposure (96 of 214 [44.9%] vs 62 of 121 [51.2%]; RR, 0.88; 95% CI, 0.70-1.10; *P* = .26), nor was IVH (77 of 197 [39.1%] vs 48 of 106 [45.3%]; RR, 0.86; 95% CI, 0.66-1.13; *P* = .29).

**Conclusions and Relevance:**

In this population-based study, findings suggest that tocolytics were associated with a reduction of death and severe IVH. Other studies are necessary to compare perinatal outcomes after use of atosiban vs CCBs.

## Key Points

**Question:**

Is tocolysis associated with lower rates of death and intraventricular hemorrhage in preterm infants owing to an effect on the fetal blood-brain barrier?

**Findings:**

In this cohort study of 1127 mothers who experienced preterm labor and delivered at gestational weeks 24 through 31, the estimated prevalence of death and/or grades III to IV intraventricular hemorrhage was significantly lower in preterm infants with vs without tocolytic exposure (25.3% vs 32.5%). Differences between atosiban and nifedipine exposure for death and/or intraventricular hemorrhage (44.9% vs 51.2%) and for intraventricular hemorrhage (39.1% vs 45.3%) were not significant.

**Meaning:**

Results for tocolytics are reassuring regarding death and/or intraventricular hemorrhage in preterm infants, but other studies appear to be necessary to compare the effects of atosiban vs nifedipine.

## Teaser

This population-based cohort study assesses the risks associated with tocolysis as measured by death and intraventricular hemorrhage in preterm infants and compares the risks between calcium channel blockers and atosiban.

## Introduction

Although no placebo-controlled studies to date have shown tocolytics to reduce neonatal mortality and morbidity, they are widely used to reduce the chance of giving birth within 48 hours.^1,2^ In very preterm infants, intraventricular hemorrhage (IVH) remains a major morbidity, observed shortly after birth and strongly associated with neonatal death or long-term neurobehavioral disabilities. At this developmental stage, IVH occurs in the vulnerable subependymal germinal matrix, characterized by intense metabolism due to neural cell precursor multiplication, immature microvessel remodeling, and sensitivity to ischemia and hemodynamic fluctuations.^3^ In France, atosiban and nifedipine are the first-line tocolytics; nicardipine hydrochloride, although not recommended, is still used in some centers.^4^ Maternal adverse effects of both compounds are well known and occur more frequently with nifedipine than atosiban.^5^ Fetal safety is more controversial, however, because both tocolytics have been detected at significant levels in fetal circulation after maternal administration.^6,7^ Nifedipine decreases uterine blood flow and fetal arterial oxygen content in pregnant sheep, and fetal deaths have been reported in humans.^8,9,10^ In contrast, calcium channel blockers (CCBs) protect the animal brain against hypoxic ischemia.^11^ Atosiban, an oxytocin receptor antagonist, could affect signaling of oxytocin receptors, whereas endogenous oxytocin appears to play a neuroprotective role in preparing the fetal brain for delivery and in newborn analgesia.^12^ We hypothesized that CCBs and/or atosiban could have different effects on IVH occurrence.

The objective of the first analysis was to estimate the association of tocolysis with death and/or IVH. In a second analysis, we compared the respective effects of both tocolytics.

## Methods

### Study Population

This study follows the Strengthening the Reporting of Observational Studies in Epidemiology (STROBE) reporting guideline for cohort analyses.^13^ EPIPAGE-2 (Enquête Épidémiologique sur les Petits Âges Gestationnels) is a prospective national population-based study scheduled to follow up preterm children born at gestational ages of 24 to 34 weeks until 12 years of age. From April 1 through December 31, 2011, recruitment took place at birth in all maternity and neonatal units of the 25 participating French regions, after the parents received information about the study and agreed to participate. Details on the protocol have been published elsewhere.^14^ The database was validated on a centralized system by the EPIPAGE national coordination team. The EPIPAGE-2 study was approved by the National Data Protection Authority, the Consultative Committee on the Treatment of Data on Personal Health for Research Purposes, and the Committees for the Protection of People Participating in Biomedical Research. Oral informed content was obtained from the participating parents.

In the present study, we included mothers who experienced preterm labor and delivered at gestational ages 24 to 31 weeks. We excluded mothers with body temperatures as high as 38.5°C, pregnancy-related vascular disease (hypertension, preeclampsia or eclampsia, HELLP [hemolysis, elevated liver enzyme levels, and low platelet count] syndrome, retroplacental hematoma, or one of these signs associated with intrauterine growth retardation), infants with lethal malformations, monozygotic twins, and fetal death in utero. Mothers with premature rupture of membranes without body temperatures as high as 38.5°C were included.

For the first analysis of the effects of tocolysis, we accounted only for the variable defined as tocolytic treatment during the last hospitalization ending in delivery. For the second analysis on the comparison of treatment (atosiban vs CCBs), we only considered mothers who received treatment within 48 hours before delivery. To analyze the effect of each drug independently, we excluded mothers who were exposed to both tocolytics.

### Diagnosis of IVH

Intraventricular hemorrhage and white matter damage were diagnosed based on cranial ultrasonography (cUS) examination findings, which were collected prospectively during hospitalization until discharge or death. At all the participating centers, qualified neonatologists or radiologists performed cUS. In France, each preterm infant undergoes 1 or 2 cUSs in the first week of life and a weekly cUS during the following 3 weeks as standard practice. The examinations occur less frequently afterward (every 2 weeks). According to the Papile classification,^15^ grade I IVH refers to germinal layer hemorrhage; grade II, IVH without ventricular dilatation; grade III, IVH with primary ventricular dilatation; and grade IV, cases of intraparenchymal hemorrhage, referring to large unilateral parenchymal hyperdensity or large unilateral porencephalic cyst possibly caused by ischemic and hemorrhagic infarctions. A composite variable was built for cerebral hemorrhages resulting in 4 mutually exclusive classes.^16^ When several cerebral lesions were successively observed, the most severe lesion was considered.

### Outcome Measures

In both analyses, the primary outcome was defined as the composite criteria of death and/or IVH. The 4 secondary outcomes were death, IVH, a composite outcome of death and/or grades III to IV IVH, and 3 classes of IVH severity that included no IVH, grade I or II IVH, and grade III or IV IVH. The death criteria included death during labor, in the maternity ward, or in the neonatal intensive care unit. For the IVH criteria, we excluded deaths during labor and in the maternity ward.

### Statistical Analysis

Data were analyzed from June 7, 2014, through September 3, 2017. In the first analysis, we compared children with vs without tocolytic exposure. The association between tocolysis and maternal characteristics, pregnancy complications, and antenatal care outcomes was first studied by univariable analysis using the Pearson χ^2^ test or the Fisher exact test as appropriate. The propensity score method was used to reduce indication bias in this study. The propensity score was defined as the participant’s probability of receiving a specific treatment conditional on the observed covariates.^17,18^ In our study, the propensity score was estimated by a multivariable logistic regression model, and 1:1 propensity score matching was adopted. The following covariates were included in the propensity score model: maternal age, geographic origin of the mother, social class of the family, tobacco consumption during pregnancy, level of neonatal intensive care associated with the maternity unit, infertility treatment, single or multiple pregnancy, antenatal magnesium sulfate use, antenatal corticosteroid use, intrauterine growth retardation, and gestational age at admission. After matching, a log-binomial model using a generalized estimation equation to account for correlations between twins was performed to compare the outcome measures between the groups with and without tocolytic exposure. A sensitivity analysis was also performed with another approach using the propensity score, namely, inverse probability of treatment weighting (after propensity score trimming to ensure comparability of preterm infants). Relative risks (RRs) and their corresponding 95% CIs were estimated.

A second analysis was performed to compare the effects of the 2 main tocolytic treatments: atosiban vs a CCB (nifedipine or nicardipine). The population used for this analysis included women who had received a single tocolytic treatment (only atosiban or a CCB) during the hospitalization that ended with delivery. We first studied the association between tocolytics and maternal complications by univariable analysis using the Pearson χ^2^ test or the Fisher exact test as appropriate. Propensity score matching and a log-binomial model using a generalized estimation equation to account for correlations between twins were performed to study associations between each tocolytic treatment and outcomes. Individual propensity score matching was performed on the following variables: maternal age, geographic origin of the mother, level of neonatal intensive care associated with the maternity unit, in utero transfer, single or multiple pregnancy, antenatal magnesium sulfate use, antenatal corticosteroid use, intrauterine growth restriction, and gestational age at admission. Two newborns whose mothers were treated with atosiban were matched to each newborn whose mother had been treated with nicardipine or nifedipine, and when this was not possible, only 1 newborn was matched. The inverse probability of the treatment weighting method (after propensity score trimming) was then used for the sensitivity analysis. For each outcome, the RRs and their 95% CIs were estimated from unadjusted log-binomial regression and after adjustment for antenatal corticosteroid use and gestational age at birth with the same model.

Statistical significance was defined as 2-sided *P* < .05. These analyses were performed with SAS software (version 9.3; SAS Institute, Inc).

## Results

The study included 1127 mothers (mean [SD] age, 25.5 [6.0] years) with preterm labor from the 2011 EPIPAGE-2 population study and 1343 infants, born at gestational ages of 24 to 31 weeks with a male to female ratio of 1.23 and a mean (SD) gestational age of 27 (2.5) weeks (Figure). Of these, 789 (70.0%) mothers received tocolytics, including 314 (39.8%) who received only atosiban and 118 (15.0%) who received only a CCB. The mean age (SD) of mothers who received tocolytics was 28.7 (5.7) years; for those who did not receive tocolytics, 28.3 (6.7) years. The rate of tocolysis treatment was significantly higher for twin gestation, high social class, infertility treatment, antenatal corticosteroid use, antenatal magnesium sulfate level, nonsmokers, or in utero transfer in a level III maternity unit (Table 1). After propensity score matching, no significant differences were observed among these variables (eFigure, A, in the Supplement), and the risk of death and/or IVH was not significantly different between preterm infants exposed to tocolytics (183 of 363 [50.4%]) and those not exposed (207 of 363 [57.0%]) (RR, 0.88; 95% CI, 0.77-1.01; *P* = .07) (Table 2). The risk of death and/or grade III or IV IVH was significantly lower in exposed infants (92 of 363 [25.3%]) than in nonexposed infants (118 of 363 [32.5%]) (RR, 0.78; 95% CI, 0.62-0.98; *P* = .03).

**Figure.**
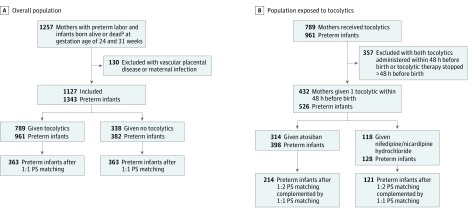
Flowcharts of the Study A, Population of mothers and preterm infants considered for the first analysis comparing groups of preterm infants with and without exposure to tocolytics. B, Population of mothers and preterm infants considered for the second analysis comparing groups of preterm infants with exposure to atosiban or calcium channel blockers. PS indicates propensity score. ^a^Indicates infants who were alive at the admission of the mother and before exposure to toxolytics and died during labor in the maternity ward before admission to or in the neonatal intensive care unit.

**Table 1.** Baseline Characteristics in the Groups of Mothers Who Received and Did Not Receive Tocolytics

Characteristic	Treatment Group, No./Total No. (%) of Mothers^a^		*P* Value
	No Tocolysis (n = 338)	Tocolysis (n = 789)	
Age, y			
<25	90/337 (26.7)	184/788 (23.4)	.35
25-34	188/337 (55.8)	475/788 (60.3)	
≥35	59/337 (17.5)	129/788 (16.4)	
Country of origin			
France or Europe	239/302 (79.1)	598/726 (82.4)	.04^b^
North African countries	28/302 (9.3)	49/726 (6.7)	
Other African countries	15/302 (5.0)	53/726 (7.3)	
Other	20/302 (6.6)	26/726 (3.6)	
Twin gestation	45/338 (13.3)	175/789 (22.2)	<.001^b^
Social class			
High	80/270 (29.6)	253/683 (37.0)	.01^b^
Medium	105/270 (38.9)	275/683 (40.3)	
Low	85/270 (31.5)	155/683 (22.7)	
Smoking	86/316 (27.2)	156/765 (20.4)	.01^b^
Infertility treatment	30/319 (9.4)	123/764 (16.1)	.01^b^
Antenatal corticosteroids (≥1 cure)^c^	60/326 (18.4)	435/773 (56.3)	<.001^b^
Birth in a level III maternity unit	208/338 (61.5)	637/789 (80.7)	<.001^b^
In utero transfer	38/336 (11.3)	406/782 (51.9)	<.001^b^
Antenatal magnesium sulfate administered	4/333 (1.2)	35/778 (4.5)	.01^b^
Gestational age at admission, wk			
<27	128/337 (38.0)	348/784 (44.4)	.03^b^
27-29	107/337 (31.8)	253/784 (32.3)	
30-31	102/337 (30.3)	183/784 (23.3)	
Intrauterine growth restriction	15/333 (4.5)	37/787 (4.7)	.86

^a^ Owing to missing data, numbers may not sum to column totals. Percentages have been rounded and may not total 100.

^b^ *P* < .05 with Pearson χ^2^ test or Fisher exact test as appropriate.

^c^ Indicates 1 complete cure with 2 injections of betamethasone at an interval of 24 hours.

**Table 2.** Outcome Measures in the Group of Infants With vs Without Tocolytic Exposure After Propensity Score Matching

Outcome	Infant Group, No./Total No. (%)		*P* Value	RR (95% CI)
	No Tocolytic Exposure (n = 363)	Tocolytic Exposure (n = 363)		
Death and/or IVH	207/363 (57.0)	183/363 (50.4)	.07	0.88 (0.77-1.01)
Death and/or grades III-IV IVH	118/363 (32.5)	92/363 (25.3)	.03^a^	0.78 (0.62-0.98)
Death	98/363 (27.0)	84/363 (23.1)	.23	0.86 (0.67-1.10)
IVH on ≥1 cUS study^b^	144/309 (46.6)	134/316 (42.4)	.29	0.91 (0.76-1.08)
If IVH, IVH in 2 classes^b^				
No IVH	165/308 (53.6)	182/314 (58.0)	.22	0.86 (0.68-1.09)
Grades I-II IVH	98/308 (31.8)	98/314 (31.2)		
Grades III-IV IVH	45/308 (14.6)	34/314 (10.8)		

Abbreviations: cUS, cranial ultrasonography; IVH, intraventricular hemorrhage; RR, relative risk.

^a^ *P* < .05 with a log-binomial model using generalized estimation equation after propensity score matching.

^b^ Excluded deaths during labor and children who died before being admitted to the neonatal intensive care unit.

Table 3 lists the baseline characteristics in the group of mothers who received atosiban and the group who received a CCB. The rate of atosiban treatment was significantly higher for women who had twin gestation (84 of 314 [26.8%] vs 11 of 118 [9.3%]; *P* < .001) or who required in utero transfer for preterm labor (171 of 314 [54.4%] vs 34 of 116 [29.3%]; *P* < .001). After propensity score matching, no significant differences were observed in these variables except for use of antenatal corticosteroids and gestational age (eFigure, B, in the Supplement), and the primary and secondary outcomes in the group of preterm infants exposed to atosiban vs the group exposed to a CCB were not significantly different, before and after adjustment for antenatal corticosteroid use and gestational age (Table 4). The risk of death and/or IVH was not significantly different between preterm infants exposed to a CCB (62 of 121 [51.2%]) and those exposed to atosiban (96 of 214 [44.9%]) (before adjustment: RR, 0.88; 95% CI, 0.70-1.10; *P* = .26; after adjustment: RR, 0.86; 95% CI, 0.72-1.03; *P* = .11). The risk of IVH was not significantly different between preterm infants exposed to a CCB (48 of 106 [45.3%]) and those exposed to atosiban (77 of 197 [39.1%]) (before adjustment: RR, 0.86; 95% CI, 0.66-1.13; *P* = .29; after adjustment: RR, 0.82; 95% CI, 0.64-1.05; *P* = .12). Sensitivity analyses using inverse probability of treatment weighting methods gave comparable results (eTables 1 and 2 in the Supplement).

**Table 3.** Baseline Characteristics in the Groups of Mothers Who Received Atosiban or a Calcium Channel Blocker

Characteristic	Treatment Group, No./Total No. (%) of Mothers^a^		*P* Value
	Nifedipine or Nicardipine Hydrochloride (n = 118)	Atosiban (n = 314)	
Age, y			
<25	25/117 (21.4)	88/314 (28.0)	.31
25-34	70/117 (59.8)	179/314 (57.0)	
≥35	22/117 (18.8)	47/314 (15.0)	
Country of origin			
France or Europe	82/109 (75.2)	240/287 (83.6)	.01^b^
North African countries	7/109 (6.4)	19/287 (6.6)	
Other African countries	17/109 (15.6)	15/287 (5.2)	
Other	3/109 (2.8)	13/287 (4.5)	
Twin gestation	11/118 (9.3)	84/314 (26.8)	<.001^b^
Social class			
High	33/106 (31.1)	87/262 (33.2)	.81
Medium	45/106 (42.5)	114/262 (43.5)	
Low	28/106 (26.4)	61/262 (23.3)	
Smoking	23/113 (20.4)	64/303 (21.1)	.88
Infertility treatment	14/115 (12.2)	49/301 (16.3)	.30
Antenatal corticosteroid use (1 cure)^c^	53/116 (45.7)	118/305 (38.7)	.19
Birth in a level III maternity unit	86/118 (72.9)	235/314 (74.8)	.68
In utero transfer	34/116 (29.3)	171/314 (54.4)	<.001^b^
Antenatal magnesium sulfate use	6/118 (5.1)	14/304 (4.6)	.81
Gestational age at admission, wk			
<27	41/117 (35.0)	136/313 (43.4)	.83
27-29	39/117 (33.3)	102/313 (32.6)	
30-31	37/117 (31.6)	75/313 (24.0)	
Intrauterine growth restriction	6/118 (5.1)	14/314 (4.4)	.77

^a^ Owing to missing data, numbers may not sum to column totals. Percentages have been rounded and may not total 100.

^b^ *P* < .05 with Pearson χ^2^ test or Fisher exact test as appropriate.

^c^ Indicates 1 complete cure with 2 injections of betamethasone at an interval of 24 hours.

**Table 4.** Outcome Measures in the Group of Infants With Atosiban vs Calcium Channel Blocker Exposure After Propensity Score Matching

Outcome	Exposure, No./Total No. (%) of Infants		*P* Value	RR (95% CI)
	Nifedipine or Nicardipine (n = 121)	Atosiban (n = 214)		
Death and/or IVH	62/121 (51.2)	96/214 (44.9)	.26^a^	0.88 (0.70-1.10)^a^
			.11^b^	0.86 (0.72-1.03)^b^
Death and/or grades III-IV IVH	26/121 (21.5)	44/214 (20.6)	.84^a^	0.96 (0.62-1.47)^a^
			.38^b^	0.85 (0.59-1.22)^b^
Death	23/121 (19.0)	41/214 (19.2)	.97^a^	1.01 (0.64-1.60)^a^
			.51^b^	0.88 (0.60-1.29)^b^
IVH on ≥1 cUS study^c^	48/106 (45.3)	77/197 (39.1)	.29^a^	0.86 (0.66-1.13)^a^
			.12^b^	0.82 (0.64-1.05)^b^
If IVH, IVH in 2 classes^c^				
No IVH	58/105 (55.2)	120/195 (61.5)	.28^a^	0.82 (0.57-1.18)^a^
Grades I-II IVH	36/105 (34.3)	58/195 (29.7)	.09^b^	0.71 (0.49-1.05)^b^
Grades III-IV IVH	11/105 (10.5)	17/195 (8.7)		

Abbreviations: cUS, cranial ultrasonography; IVH, intraventricular hemorrhage; RR, relative risk.

^a^ Log-binomial model using generalized estimation equation without adjustment after propensity score matching.

^b^ Log-binomial model using generalized estimation equation with adjustment for at least 1 complete corticosteroid cure and gestational age (≤28 and >28 weeks) after propensity score matching.

^c^ Excluded deaths during labor and children who died before being admitted to the neonatal intensive care unit.

## Discussion

In this EPIPAGE-2 study, rates of death and severe grades III to IV IVH, a secondary end point, were reduced among infants born after preterm labor and whose mothers received tocolytic treatment. Although a similar trend was observed for the primary end point, death and/or IVH, the association was not significant. Among those exposed to tocolytics, nonsignificant differences in death and/or IVH and in secondary outcomes were observed between infants exposed to atosiban and those exposed to a CCB.

In perinatal medicine, balancing the benefits and harms of drugs administered to women at risk of very preterm births remains a challenge for obstetricians and neonatologists. Tocolytics do not act specifically on the uterus and cross the placenta to the fetus.^5,6^ Accordingly, they have multiorgan effects and may deviate molecular pathways leading to mature functional blood-brain barrier and neuronal circuits.^1,4^ In this EPIPAGE-2 population study, we did not observe an increased risk of death and/or IVH, suggesting that tocolysis with CCBs or atosiban is safe for this specific outcome. This observation is of clinical importance and is reassuring regarding the recommendation to use a tocolytic, either nifedipine or atosiban, during the first 48 hours of preterm labor before a gestational age of 32 weeks to prolong gestation, achieve fetal lung maturation by fluorinated corticosteroid administration, and protect the fetal brain by magnesium sulfate infusion.^19,20^

In our study, we observed an absence of significant differences in rates of death and/or IVH, death and/or severe IVH, death, and IVH rates between atosiban and CCBs. Preclinical studies indicated that nifedipine and atosiban have various effects, in particular in the developing central nervous system, justifying comparative clinical research to control their respective adverse effects. Nifedipine is a long-lasting CCB of the dihydropyridine family, widely identified in cerebral arteries and in dendritic spines, which control neuronal excitability and plasticity of glutamatergic synapses as well as paracellular permeability of the blood-brain barrier.^11,21,22^ In an in vitro blood-brain barrier system, a hypoxic or a hypoxic-aglycemic stress induced a disruption of the blood-brain barrier, which was prevented by a paradoxical increase in the intracellular calcium level after nifedipine treatment. Nifedipine has a neuroprotective effect in cultured neurons against glutamate-induced damage and induces stabilization in intracerebral blood pressure.^21,22^ By contrast, it has deleterious effects on fetal and maternal hemodynamics in pregnant sheep.^8^

Atosiban is an antagonist of oxytocin, a neurotransmitter involved early in neuroprotection, social function, and cognition during brain development.^23,24^ Birth is associated with an oxytocin-mediated, dramatic, abrupt, and short-lasting reduction in chloride, which has neuroprotective action on the newborn. Maternal administration of a selective receptor antagonist shortly before birth could prevent this shift in the neurons of the offspring, increasing susceptibility to anoxia and IVH.^25^ In the latest update of the Cochrane reviews on atosiban for inhibiting preterm labor,^26^ no superiority of atosiban was observed compared with CCBs in terms of pregnancy prolongation or extremely preterm birth. However, the review was limited, because only 2 studies of poor methodologic quality (no randomization and bias of indication) including 225 women were considered. Moreover, no data were available for perinatal mortality. In the recent APOSTEL III (Nifedipine vs Atosiban for Threatened Preterm Birth) randomized clinical trial comparing nifedipine vs atosiban for preterm labor at a gestational age of 25 to 34 weeks,^27^ 48 hours of tocolysis resulted in similar perinatal composite outcomes, including perinatal mortality, sepsis, periventricular leukomalacia, grade III or IV IVH, necrotizing enterocolitis, and chronic lung disease. A nonsignificant increase in the rates of grade III or IV IVH (2% vs 1%; RR, 2.47; 95% CI, 0.48-12.75) and perinatal mortality (5% vs 2%; RR, 2.20; 95% CI, 0.91-5.33) were observed with nifedipine. These results are similar to our findings of a nonsignificant increase in the rates of death and/or IVH, IVH, and severe IVH with administration of a CCB (51.2%, 45.3%, and 10.5%, respectively) compared with atosiban (44.9%, 39.1%, and 8.7%, respectively). In a smaller secondary APOSTEL III study of 117 children (51 exposed to nifedipine and 66 to atosiban) born before a gestational age of 32 weeks, rates of severe brain injury were not different between both tocolytic groups, but this secondary analysis of the trial was underpowered.^28^ Altogether these studies do not allow strong conclusions on the superiority of one type of tocolytic over another. An uncertainty that CCBs have more neonatal adverse effects (death and IVH) than atosiban remains.

### Strengths and Limitations

This EPIPAGE-2 study is a large population-based prospective cohort study including all the preterm births at a gestational age of 22 to 34 weeks occurring in 25 of the 26 regions of France during 8 months in 2011. The double validation of cUS and the analysis by the same group of neonatologists who reviewed all cases of grades III and IV IVH reduce the risk of intraobserver variability and add to its power.^16^ However, in this study, tocolytics were not administered randomly, but might have been determined by maternal characteristics and indications that made the 2 groups (treated and not treated) different. To control for the potential indication bias, we used a propensity score analysis and adjusted for confounding factors, thus minimizing the probability of incorrectly attributing the association of death and/or IVH to tocolytic administration. However, after matching, some differences persist in some of the variables, necessitating additional adjustments and limiting the study. Moreover, we accounted for the correlations of outcomes for twins of the same mother through a generalized estimation equation approach within propensity score matching and performed a sensitivity analysis by using inverse probability of treatment weighting. Another limitation of our study could be that the available sample size after matching was too small to detect a clinically meaningful difference.

## Conclusions

Tocolysis in women in preterm labor is associated with a decrease in death or severe IVH in preterm infants, suggesting that tocolysis is safe and may even have neonatal benefits. Other short-term neonatal studies and school-age cognitive and behavioral follow-up studies with a higher number of preterm infants exposed to atosiban or CCBs are urgently needed to devise guidelines for better tocolytic strategies to optimize preterm infant brain development.

## Notes

Supplement. **Table 1.** Outcome Measures After IPTW in the Group of Infants Exposed to Tocolytics vs the Group of Infants Not Exposed **Table 2.** Outcome Measures After IPTW in the Group of Infants Exposed to Atosiban vs the Group of Infants Exposed to a Calcium Channel Blocker **eFigure.** Standardized Difference of Maternal Covariates Before and After Matching
