# Supplementary material for: Association of Intraventricular Hemorrhage and Death With Tocolytic Exposure in Preterm Infants
Source: JAMA Netw Open. 2018 Sep 21;1(5):e182355. doi: 10.1001/jamanetworkopen.2018.2355 (PMC6324618; doi:10.1001/jamanetworkopen.2018.2355)
Supplement: Supplement. — Table 1. Outcome Measures After IPTW in the Group of Infants Exposed to Tocolytics vs the Group of Infants Not Exposed Table 2. Outcome Measures After IPTW in the Group of Infants Exposed to Atosiban vs the Group of Infants Exposed to a Calcium Channel Blocker eFigure. Standardized Difference of Maternal Covariates Before and After Matching [file jamanetwopen-1-e182355-s001.pdf]

Supplementary Online Content

Pinto-Cardoso G, Houivet E, Marchand-Martin L, et al; EPIPAGE-2 Working Group. Association of intraventricular hemorrhage and death with tocolytic exposure in preterm infants. *JAMA Netw Open.* 2018;1(5):e182355. doi:10.1001/jamanetworkopen.2018.2355

Table 1. Outcome Measures After IPTW in the Group of Infants Exposed to Tocolytics vs the Group of Infants Not Exposed

Table 2. Outcome Measures After IPTW in the Group of Infants Exposed to Atosiban vs the Group of Infants Exposed to a Calcium Channel Blocker

eFigure. Standardized Difference of Maternal Covariates Before and After Matching

This supplementary material has been provided by the authors to give readers additional information about their work.

©Pinto-Cardoso G et al. *JAMA Network Open*

Table 1. Outcome Measures After IPTW in the Group of Infants Exposed to Tocolytics vs the Group of Infants not Exposed

	No tocolysis	Tocolysis		
	***n/N* (%)**	***n/N* (%)**	***P* Value**	**RR (95% CI)**
**Death and/or IVH**	202/346 (58.4)	370/791 (46.8)	.06	0.86 (0.73-1.01)
**Death and/or grade III- IV IVH**	115/346 (33.2)	167/791 (21.1)	**.02***	**0.72 (0.55-0.94)**
**Death**	96/346 (27.8)	145/791 (18.3)	.30	0.86 (0.65-1.15)
**IVH on at least one cUS†**	142/295 (48.1)	293/722 (40.6)	.17	0.87 (0.71-1.06)
**If IVH, IVH in two classes†**				
No IVH	153/294 (52.0)	429/719 (59.7)	.11	0.80 (0.60-1.05)
Grade I- II IVH	95/294 (32.3)	218/719 (30.3)		
Grade III- IV IVH	46/294 (15.7)	72/719 (10.0)		

Data are *n/N* (%) unless otherwise specified
cUS: cranial ultrasonographic study; IPTW: inverse probability of treatment weighting; IVH: intraventricular hemorrhage
† Excluded deaths during labor and children who died before being admitted to NICU
*p < 0.05 with log-binomial model using generalized estimation equation with IPTW (after PS trimming)

©Pinto-Cardoso G et al. *JAMA Network Open*

Table 2. Outcome Measures After IPTW in the Group of Infants Exposed to Atosiban vs the Group of Infants Exposed to a Calcium Channel Blocker

	**Nifedipine or Nicardipine**	**Atosiban**		
	***n/N* (%)**	***n/N* %**	***P* Value**	**RR (95% CI)**
**Death and/or IVH**	64/124 (51.6)	177/377 (46.9)	.89[a]<br>.64[b]	1.03 (0.70-1.52)[a]<br>0.95 (0.77-1.17)[b]
**Death and/or grade III- IV IVH**	28/124 (22.6)	81/377 (21.5)	.77[a]<br>.93[b]	1.09 (0.61-1.93)[a]<br>0.98 (0.65-1.47)[b]
**Death**	25/124 (20.2)	69/377 (18.3)	.84[a]<br>.89[b]	1.07 (0.58-1.96)[a]<br>0.97 (0.62-1.51)[b]
**IVH on at least one cUS**[†]	48/108 (44.4)	141/348 (40.5)	.93[a]<br>.57[b]	1.02 (0.65-1.61)[a]<br>0.91 (0.67-1.25)[b]
**If IVH, IVH in two classes**[†]				
No IVH	60/107 (56.1)	206/345(59.7)	.94[a]	1.03 (0.57-1.85)[a]
Grade I- II IVH	36/107 (33.6)	104/345 (30.1)	.67[b]	0.89 (0.52-1.52)[b]
Grade III- IV IVH	11/107 (10.3)	35/345 (10.2)		

Data are *n/N* (%) unless otherwise specified
cUS: cranial ultrasonographic study; IPTW: inverse probability of treatment weighting; IVH: intraventricular hemorrhage
[a] Log-binomial model using generalized estimation equation with IPTW (after PS trimming)
[b] Log-binomial model using generalized estimation equation with IPTW (after PS trimming) and adjustment for at least one complete corticosteroid cure and gestational age (≤28 and >28)
[†] Excluded deaths during labor and children who died before being admitted to NICU

eFigure. Standardized Difference of Maternal Covariates Before and After Matching

A. First analysis comparing groups of infants exposed to tocolysis or not

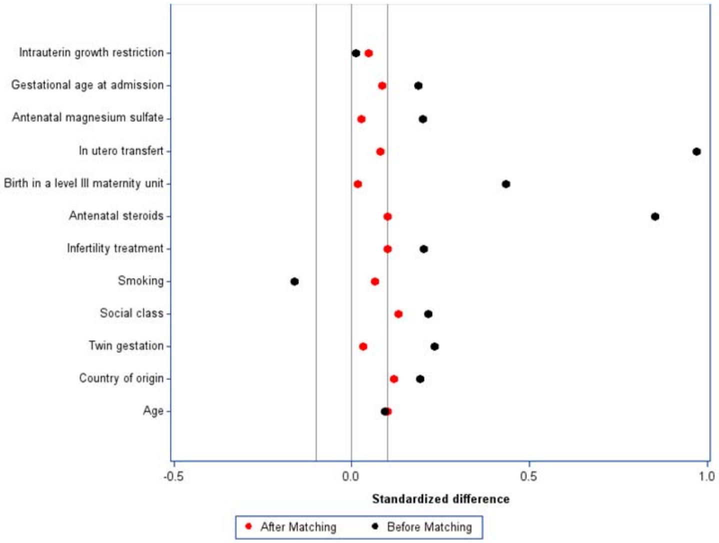

B. Second analysis comparing groups of infants exposed to atosiban or to calcium channel blockers

©Pinto-Cardoso G et al. *JAMA Network Open*

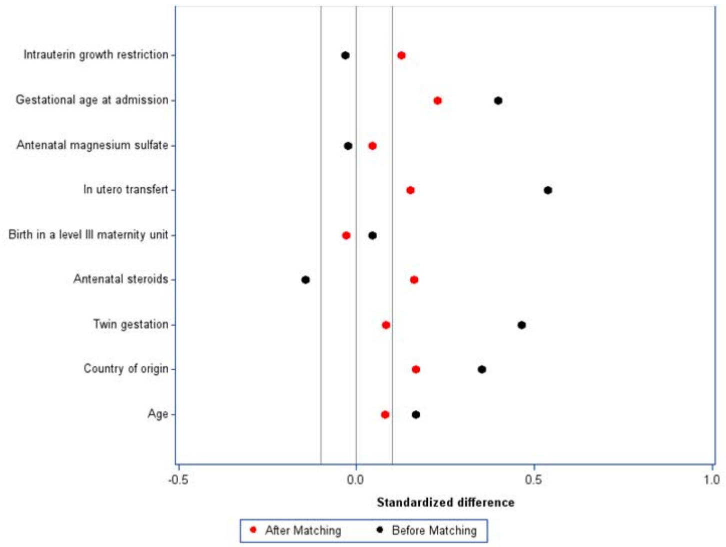

©Pinto-Cardoso G et al. *JAMA Network Open*
